# Standardization of surface electromyography utilized to evaluate patients with dysphagia

**DOI:** 10.1186/1746-160X-3-26

**Published:** 2007-06-06

**Authors:** Michael Vaiman

**Affiliations:** 1 Department of Otolaryngology, Assaf Harofe Medical Center, Affiliated to the Sackler Faculty of Medicine, Tel Aviv University, Tel Aviv, Israel; 2 33 Shapiro Street, Bat Yam, 59561, Israel

## Abstract

**Backgorund:**

Patients suspected of having swallowing disorders, could highly benefit from simple diagnostic screening before being referred to specialist evaluations. We introduce surface electromyography (sEMG) to carry out rapid assessment of such patients and propose suggestions for standardizing sEMGs in order to identify abnormal deglutition.

**Methods:**

Specifics steps for establishing standards for applying the technique for screening purposes (e.g., evaluation of specific muscles), the requirements for diagnostic sEMG equipment, the sEMG technique itself, and defining the tests suitable for assessing deglutition (e.g., saliva, normal, and excessive swallows and uninterrupted drinking of water) are presented in detail. A previously described normative database for single swallowing and drinking and standard approach to analysis was compared to data on the duration and electric activity of muscles involved in deglutition and with sEMG recordings in order to estimate stages of a swallow.

**Conclusion:**

SEMG of swallowing is a simple and reliable method for screening and preliminary differentiation among dysphagia and odynophagia of various origins. This noninvasive radiation-free examination has a low level of discomfort, and is simple, timesaving and inexpensive to perform. With standardization of the technique and an established normative database, sEMG can serve as a reliable screening method for optimal patient management.

## Background

Swallowing disorders (dysphagia) occurs in approximately 14% of patients in acute care setting an up to 50% of patients in nursing homes [1]. Its prevalence is related to the fact that dysphagia often is present in patients who have sudden-onset neurologic disorders, chronic neurodegenerative diseases, and patients with general medical problems, but in general it is an interdisciplinary phenomenon. It is frequently associated with painful swallowing, or odynophagia. It is estimated that 15 million patients suffered from a swallowing disorder during each year in the United States alone [2,3]. Patients with suspected dysphagia and/or odynophagia could highly benefit from preliminary screening for confirmation of diagnosis before being referred for more extensive clinical and instrumental evaluations by a specialist. Currently videofluorographic swallow study (VFSS) is the most commonly used tool in the assessment of oropharyngeal dysphagia, and it is considered the gold standard in the dysphagia workup. Unfortunately, VFSS has several drawbacks, as the patient must be transported to the radiology suite, must be able to cooperate with the examination, and will be exposed to radiation. Also, VFSS does not always identify neuromuscular abnormalities in pharyngeal or laryngeal physiology. A reliable, noninvasive, time-saving and inexpensive procedure that might be easily learned and applied by primary health clinicians as well as by nurses would be a valuable addition to our diagnostic armamentarium.

Given that the swallowing mechanism by which food is transmitted to the stomach is a complex action involving 26 muscles and five cranial nerves, electromyography (EMG) would appear especially suitable for screening and early diagnosis of dysphagia and odynophagia. Indeed, surface EMG (sEMG) provides information on the timing of selected muscle contraction patterns during swallowing [4-6], on the amplitude of electric activity of the muscles [7], and was shown to be easily learned by medical personnel [8,9]. EMG had already been proposed for screening purposes in neurogenic dysphagia [10].

For the past five years, we have been investigating deglutition by means of sEMG. Numerous studies on EMG activity of face and neck muscles during swallowing had appeared in the 1990s [3-11] and revealed a lack of agreement among experts regarding some of the basic aspects of the act of swallowing common to all subjects as well as in differentiating between the values that represent normal and abnormal function. Thus, we first established a normative database for deglutition for adults [12,13] and children [14] and now embarked on devising standards for sEMG in diagnosing it. It emerged that there is a large variation in examination techniques, strategies, interpretations and diagnostic criteria among electromyographers [15], further reinforcing the need for international standardization.

In the current article, we introduce sEMG as a rapid screening method for patients with complaints suggestive of dysphagia or odynophagia that need to be differentiated and localized in oral, laryngeal and esophageal causes. We also suggest steps for standardization of sEMG assessment of normal and abnormal deglutition, as had been done for electrocardiograms one hundred years ago. Indeed, as patients with chest pains are more likely to approach their primary health provider before consulting a cardiologist, we expect patients with swallowing disorders to be seen first by family physicians before consulting an otolaryngologist.

## Standardization of the diagnostic procedure

Any diagnostic method designed for use in different areas of medicine requires standards, and we propose the following for EMG evaluation of deglutition

### Standards for test application

Ever since Magendie's publications in 1813, physicians have adapted the concept of three stages in swallowing, oral, pharyngeal, and esophageal [16,17]. This was altered to four stages in the 1980s, with the oral stage having been divided into oral initial (for solids, the "oral preparation" stage) and oral final stages [18]. This latter staging can be helpful in diagnosing disorders that lead to dysphagia and odynophagia. In liquid swallowing, the water intake takes place during the oral initial stage starting with sealing of the labia. The oral final stage occurs when the tongue squeezes the liquid volume against the hard palate so that it is propelled past the anterior faucial arches, whereupon the automatic reflexive gesture of swallowing is triggered. During the pharyngeal stage, the liquid volume is transferred from the level of the faucial arches through the pharynx to the cricopharyngeal sphincter at the rostral aspect of the esophagus. In the esophageal stage of the swallow, the water volume is transferred in a continuation of the peristaltic movement from the cricopharyngeal to the gastroesophageal sphincter at the entrance to the stomach.

Each of these stages can be impaired and the screening evaluation should be capable of indicating which is the impaired stage. (Surface EMG recordings cannot trace esophageal activity, only the initial esophageal stage.)

### Standards for the equipment

To carry out rapid and accurate assessments, the diagnostic tool should be reliable, preferably noninvasive, preferably radiation-free, inexpensive, time saving and simple and easy to operate. Surface EMG devices meet all these criteria. We propose a fourchannel computerbased EMG unit equipped with surface electrodes. We use standard surface electrodes AE-131 and AE-178 which are silvercoated discs with 11 mm diameters and placed 10 mm from each other. Other surface electrodes with similar characteristics can be used as well. In our earlier studies, sEMG recordings were performed by a NeuroDyne Neuromuscular Sys/3 fourchannel computerbased EMG unit with NeuroDyne Medical software (NeuroDyne, Cambridge, MA, USA) and AE-204 active sensors attached to AE-131 or AE-178 electrodes [12].

Any other EMG device with similar characteristics can be used as long as the EMG recording is full-wave rectified and low-passed filtered in such a way that it resembles a single EKG line. EMG records with numerous closely packed spikes are almost impossible to interpret rapidly. A 2-channel EMG is not enough for rapid testing, and 8-channel EMG records are difficult to perform and take a considerable amount of time to interpret: testing with the 4 channel device can take only 5–7 minutes when the patient is fully cooperative.

### Standards for the electromyographic technique

The four examined muscle groups are the superior and inferior orbicularis oris (OO), the masseter (MS), the submental muscle (SUB) group, which includes the anterior belly of the digastric, mylohyoid, and geniohyoid, and the infrahyoid group (INF), which includes also the laryngeal strap muscles and the thyrohyoid, all covered by the platysma. These muscles are superficial and are thought to be involved in the oral and pharyngeal phases of a swallow.

**Figure 1:**
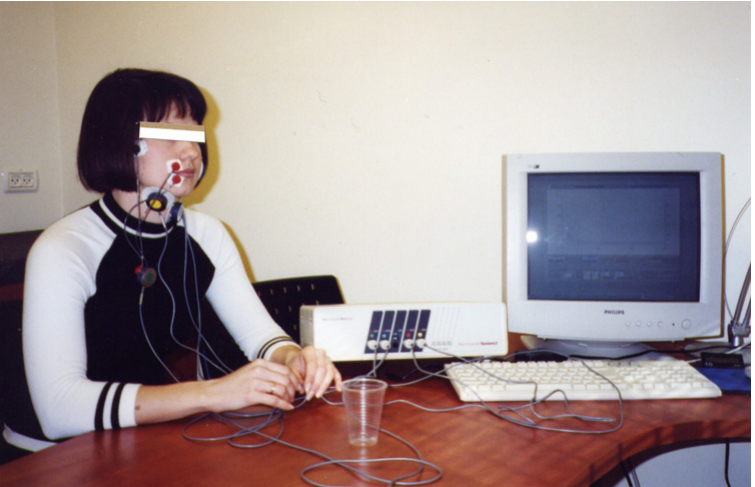
The electroneuromyograph NS/3 in operation.

The suggested standard electrode positions are as follows (Fig. 1):

1. Two bipolar stick-on surface electrodes applied at the right or left angle of the mouth, one electrode above the upper lip, and another electrode below the lower lip (OO-location);

2. Two electrodes parallel to the MS fibers on the left or right side of the face, preferably on the opposite side from the OO-location (MS-location);

3. Two surface electrodes on the skin beneath the chin on the right or left side of midline to record SUB myoelectrical activity over the platysma (SUB-location);

4. Two electrodes on the left or right side of the thyroid cartilage to record from the laryngeal strap and infrahyoid muscles (INF-location).

The exact electrode positions for each muscle group have been known since the 19^th ^century [20,21], and can be adjusted to accommodate anatomical exceptions [22]. Each pair of electrodes has a third electrode as ground.

### Standards for testing procedures (Fig. 2)

**Figure 2:**
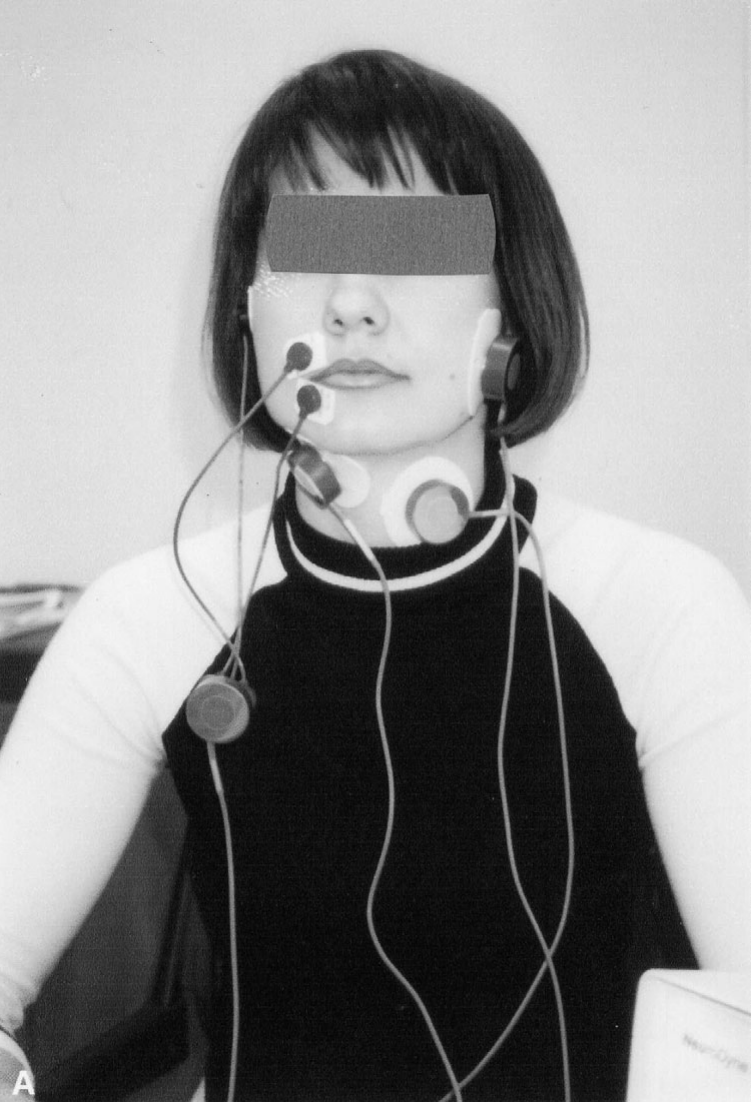
Locations of electromyogram electrodes for testing orbicularis oris (OO), masseter (MS), submental group (SUB) and laryngeal strap muscles (INF).

The proposed set of four tests includes voluntary single swallows of saliva ("dry" swallow), voluntary single water swallows from an open cup ("normal"), voluntary single swallows of an excessive amount of water (20 ml, "stress test"), and continuous drinking of 100 cc of tap water from an open cup. Subjects are permitted to move their chins slightly upwards while swallowing if needed when it emerges that there is no changes of the graphic and numerical baseline associated with this movement. (This movement involves the mm. rectus capitis posterior minor and minor, as well as some other posterior neck muscles, and does not affect signals from the abovementioned electrode locations.) The tasks to be performed are:

1. Three trials of "dry" swallowing. Instruction: "Swallow your saliva".

2. Three trials of swallowing normal volume of tap water with a mean volume of 16.5 cc. Instruction: "Swallow once in your usual way".

3. Three trials of swallowing 20 cc of tap water to check adaptation abilities of the patients ("stress test", larger bolus volume accommodation). Instructions: "Swallow in one gulp".

4. One trial of continuous drinking of 100 ml of tap water. Instruction: "Drink this in your usual way".

### Normative database and standards for analysis

**Figure 3:**
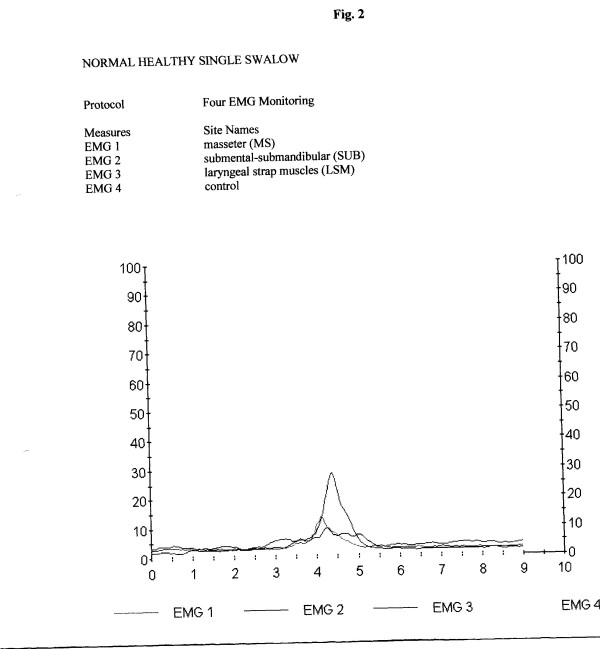
Stages of the normal swallow (reflex part). Horizontal mark 3 – water intake, 3.5–4 – final oral stage, 4–4.5 – pharyngeal stage, 4.5–5.5 – initial esophageal stage. Upper peak – submental location, middle peak – masseter location, lower peak – infrahyoid muscles location. Total electric activity duration after water intake: 2.5 sec.

**Figure 4:**
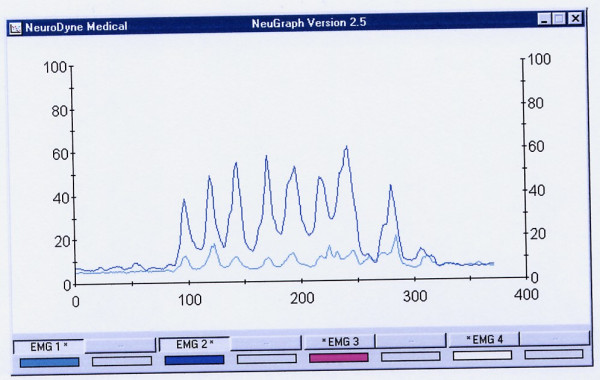
An example of normal drinking of 100 cc of water. It took this subject 15.04 sec to drink 100 cc of water in 7 swallows. The last 8^th ^peak is a dry swallow aftereffect. Upper line = the submentalsubmandibular electrode location, lower line = the masseter location. All these muscles are almost completely relaxed before and after drinking.

Standards for analysis include assessment of duration (in sec) of the swallowing act, amplitude of electric activity (mean, in μV), graphic patterns and number of swallows (in the continuous drinking task) (Tables 1 and 2). A normative database for these variables in adults [12] and children [14] was introduced earlier. (Figs. 3 and 4).

**Table 1:** Simplified set of normative data for timing measures in reflex normal swallowing (oral final stage + pharyngeal stage + initial esophageal stage) [age range: duration range, sec]

Age groups (*y*)			
Saliva swallow	18–70: **1.0–5.44**		70+: **1.44–6.24**
Normal swallow	18–70: **1.0–5.74**		70+: **2.3–6.7**
20 cc swallow	18–70: **1.8–6.2**		70+: **1.8–8.13**
One swallow while drinking	18–70: **0.56–2.56**		70+: **0.55–3.0**
100 cc drinking	18–60: **6.2–15.4**	61–70: **5.4–21.4**	70+: **7.1–28.3**
# swallows while drinking	18–40: **4–9**	41–70: **4–12**	70+: **4–16**

**Table 2:** Quick reference simplified set of normative data for electric activity obtained by surface EMG for the masseter, submental group (SUB) and laryngeal strap muscles (INF) during various tests, in μV. [age range: normal range of voltage values]

**Saliva swallow**			
Masseter range	18–30: **4.5 – 15.9**	31–70: **5.54 – 12.1**	70+: **2.94–22.42**
SUB range	18–30: **13.4–59.72**	31–70: **9.52 – 49.5**	70+: **10.2–42.32**
INF range	18–40: **2.0–4.5**	41–70: **2.5–4.3**	70+: **4.3–7.25**
**Normal swallow**			
Masseter range	18–60: **2.2–31.0**	61–70: **1.97 – 27.69**	70+: **3.77–20.0**
SUB range	18–30: **11.4–63.41**	31–50: **12.58–51.6**	51–70+: **7.4 – 44.8**
INF range	18–40: **2.85–6.3**	41–70: **3.8–5.6**	70+: **4.33–8.0**
**Excessive swallow**			
**M**asseter range	18–40:**1.5–37.0**	41–70: **1.2 – 29.4**	70+: **4.65–21.13**
SUB range	18–30: **19.28–50.80**	31–70+: **12.1 – 47.44**	
INF range	18–40: **3.8–6.55**	41–70: **3.9–6.23**	70+: **4.5–9.3**
**100 cc drinking**			
Masseter mean (real)*	18–70: **0.8 – 6.2**		70+: **1.0 – 7.84**
SUB mean (real)	18–60: **3.5 – 11.5**	61 – 70+: **4.25 – 16.25**	
INF mean (real)	18–70: **1.4–2.8**		70+: **1.0–3.85**

*Raw mean = computer-calculated mean, while Real mean = raw mean minus the mean resting potential of an actual muscle covered by skin (2.808 μV = for the m. submental and m. infrahyoid, 2.495 μV = for the m. masseter, and 4.542 = for the m. orbicularis oris).

There was no visible difference between the shapes of EMG recordings of swallows based on gender [12,13]. Elderly patients (aged 70+ years) showed age-related peculiarities in the recorded swallows. For them, the muscle activity is usually longer in duration, and suggests a lack of coordination between activities of different muscles involved in deglutition. For children, the duration of muscle activity during swallows and drinking in all tests decreased significantly with age [14]. There was, however, no statistically significant difference in electric amplitude measurements between children and adults (p = 0.05).

## Discussion

Swallowing disorders comprise an interdisciplinary phenomenon. Practitioners in various fields of medicine, such as otorhinolaryngology, neurology, general medicine, gastroenterology, head and neck surgery, dentistry and facial surgery, pediatrics and psychiatry deal with these disorders regularly, but family doctors and emergency department personnel might well be the first physicians to evaluate these patients. The need for established standards in various EMG investigations is well recognized [23-25], and several attempts already have been made to determine them [26,27]. To our knowledge, no such standards were proposed for sEMG evaluations of dysphagia and odynophagia. While an EMG evaluation of deglutition is not a new diagnostic method, the lack of standard requirements negatively impacts the value of this investigative technique. The objectives of the current work were to suggest potential solutions to this drawback by determining optimal sEMG standards suitable for investigation of face and neck muscles involved in deglutition, to establish a normal database, and to set down investigative standards to differentiate between specific pathological EMG patterns of abnormal deglutition.

### EMG records

To carry out the rapid assessment of patients, the EMG record should be clear and easily understandable, and we again stress a need for filtering EMG recordings. A recent comprehensive study in which sEMG was used for monitoring functionally distinct muscle activation during swallowing [27] supports our contention that raw sEMG records should be rectified and filtered before evaluation.

### EMG electrode locations

The proposed electrode locations were chosen in order to cover all stages of a swallow. The staging of normal deglutition can be clinically important as an additional tool for establishing etiology and localization (oral, pharyngeal, or esophageal) of the causes of dysphagia or odynophagia. Each stage has its mean normal duration and its specific graphic pattern. While additional research is needed, our preliminary assumptions are that OO and MS electrode locations represent the initial oral stage of swallowing, that the MS and SUB locations represent the final oral stage, that the MS, SUB and INF locations are important for evaluating the pharyngeal stage, and that the SUB and INF locations represent the initial esophageal stage. Therefore, when the reflex part of a swallow is investigated in healthy volunteers, the OO location provides less informative data and can be safely ignored. This location, however, might be important for patients with dysphagia due to abnormal eating patterns, problems with dentition, congenital abnormalities of the nasal and oral cavity, and others. In these cases, even without reliable normative data, the evaluation of OO sEMG activity might be important if different tests are compared within the same patient.

### Tests

The fundamental test is a single swallow of water in a normal manner. Saliva swallow test is especially relevant in cases of salivary gland diseases, such as Sjögren syndrome [28]. The stress test with an excessive amount of water swallowed in one gulp might reveal a lack of larger bolus volume accommodation abilities in cases of anatomical changes of the pharynx or neurological problems. Testing of continuous drinking is important not only in the evaluation of dysphagia but also of odynophagia and in the differential diagnosis in cases of compulsive water drinking, excessive water drinking, the malingering of dysphagia and psychogenic disorders expressed by symptoms of dysphagia. This test is most suitable for cases of mild dysphagia in easily tiring subjects for whom continuous non-interrupted drinking is a stress test. The amount of water for continuous drinking test was set at 100 cc, i.e., approximately onehalf of a standard glass, because a smaller volume, e.g., 50 cc, can be swallowed in two gulps and thus yield inadequate data while 200 cc of water involves considerable swallowing/ventilation interactions which can confound the validity of the obtained data.

### Duration of a swallow

**Figure 5:**
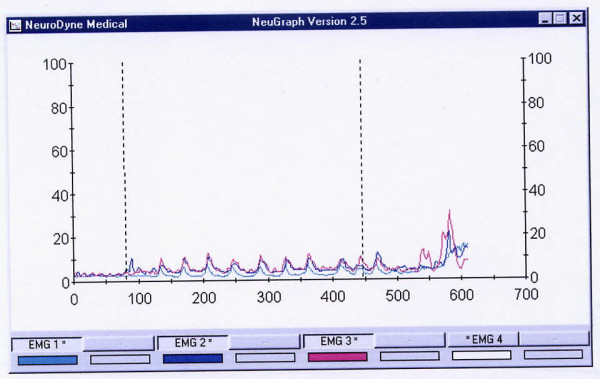
A prolonged drinking of a 75 year old subject suffering with influenza (MS = green line, SUB = blue line, and INF = red line locations). It took this subject 46 sec to drink 100 cc of water in 11 swallows. The electric amplitude is characteristically low. INF muscles are more involved in swallowing than usual, thus SUB and INF lines are almost identical.

Older people (aged 70+ years) swallow and drink more slowly as do patients with various neurological disorders affecting deglutition. (Fig. 5) The times indicated by an EMG device represent the duration of sEMG activity which lasted longer than the actual time required to pass a bolus from the oral cavity to the esophagus.

### Electric amplitude

The range (amplitude, in μV) and mean of electric activity are less important for stage-by-stage evaluation of a sEMG recording. These data might be useful, however, when abnormal swallows are investigated. For example, a person usually presents low electric activity at the MS location after undergoing a tooth extraction. We also observed patients with a Zenker diverticulum [29] who presented unusually high electric activity at the INF location, and numerous patients with recurrent tonsillitis or during flu with abnormally high electric activity of their infrahyoid muscles. (Fig. 4). There are also numerous reports on changes of the MS electric activity in patients with diseases of the temporomandibular joint [30,31].

Published studies on normal subjects show a very wide range of normal electric amplitudes for sEMG studies. These variations are not only due to biologic causes but are also greatly affected by such technical factors as skin/electrode impedance, depth of the muscle from the skin surface, location of the recording electrodes in relation to anatomic structures, variation in muscle size among individuals, and temperature. It is because of the wide variation in the normal values that an absolute value of the amplitude is considered less clinically useful. While we feel that the amplitude data might be valuable for comparison across subjects, some additional information is needed to clarify this issue. The EMG amplitude, however, remains an important aspect in the relationship between muscle force and the associated electric activity, although there is no simple relationship between a sEMG signal and muscle force. When all the different types of neuromuscular disorders are considered collectively, amplitudes are by far the most informative features. Indeed, some authors argue that amplitudes are the only components that have a direct relationship to clinical symptoms (muscle weakness) in neurogenic lesions [33].

During sEMG testing, there is a certain amount of impedance noise that arises directly from the resistance of the electrodes' connection to the skin. This feature makes skin resistance a significant factor when working with the lowlevel EMG signals typical of the small muscles involved in swallowing. Wiping the skin with isopropyl alcohol in a water solution has proven to be the best form of preparation for most situations. The alcohol removes the dead skin and surface oils, and the water moistens the skin and provides improved ion flow. The sEMG sensors we used are designed so that the use of electrode gel is generally not necessary.

Dysphagia is a very common finding in patients with neurologic disturbances like amyotrophic lateral sclerosis, Parkinson's disease, Huntington's disease, Multiple sclerosis, myasthenia gravis, stroke, laryngeal nerve injury and many others. This study emphasizes that a sEMG analysis of all the muscle groups involved in swallowing process, following a proper placement of the electrodes and selection of tests, can give reliable indications of muscle activity and provides data for screening evaluation of complaints a patient came with. Further investigation might help to develop a proper combination of flexible endoscopic evaluation of swallowing (FEES) with a nasopharyngoscope (or flexible endoscopic evaluation of swallowing with sensory testing, FEESST) [34] with SEMG to achieve complete evaluation of swallowing without exposure to radiation. Such screening might help a general practitioner to direct a patient to a neurologist, otolaryngologist or other proper specialist and to a specific radiographic or, perhaps, manofluorographic investigation.

## Conclusion

Surface EMG of swallowing is a simple and reliable method for screening and initial evaluation of dysphagia and odynophagia complaints of various origins. This noninvasive radiation-free examination has low level of discomfort, and is simple, time-saving and inexpensive. With proper standard technique and established normative database, sEMG can serve as a reliable screening method for the assessment of dysphagia of unknown origin for optimal patient management.
